# The Bad Side of a Good Economy: How Perceptions of the Economy Moderate the Relationship between Financial Strain and Powerlessness

**DOI:** 10.1177/01902725261421204

**Published:** 2026-04-05

**Authors:** Jiarui (Bruce) Liang, Alexander Wilson, Scott Schieman

**Affiliations:** 1 Yale University, New Haven, CT, USA; 2 University of Toronto, Toronto, Canada

**Keywords:** economic perceptions, financial strain, powerlessness, U.S.-Canada comparison, vibecession

## Abstract

How do perceptions of the economy moderate the association between financial strain and powerlessness? This article tests four novel hypotheses using two nationally representative samples of American (N = 2,466) and Canadian (N = 2,501) workers collected in late 2023. The amplified threat and protective economic optimism hypotheses suggest that for those who are financially struggling, perceiving the economy as “poor” is associated with more powerlessness and perceiving it as “good” is associated with less powerlessness, respectively, relative to those who perceive the economy as “fair”; the comparison-protection and meritocratic attribution hypotheses propose the opposite. We find support for the meritocratic attribution hypothesis in both countries. Those who are financially struggling report higher levels of powerlessness if they perceive a good economy than if they perceive a poor or fair economy. In other words, the positive association between personal financial strain and powerlessness is stronger among those who perceive a good economy. By contrast, we do not find evidence that perceiving a poor economy weakens financial strain's association with powerlessness relative to those who perceive a fair economy.

Public perceptions about the state of the economy have occupied media headlines in recent years. Multiple surveys in the United States and Canada have documented what many observers identify as persistent unfavorable evaluations of the economy (Amanat 2023; Casselman and DePillis 2024; Schieman, Wilson, and Liang 2023). For example, surveys find that 62 percent of Canadians describe national economic conditions as either “bad” or “very bad” (Amanat 2023) and that 51 percent of Americans characterize the U.S. economy as “poor” (Casselman and DePillis 2024). Some have observed that these negative perceptions have persisted despite contradictory evidence on key objective economic indicators (Krugman 2024b). This has inspired arguments that public perceptions about the economy—popularly referred to as “bad vibes”—are not grounded in objective reality (e.g., Sahm 2023). These bad vibes may not even be grounded in people's personal financial conditions given that surveys indicate that more than 50 percent of Americans are optimistic about their own personal financial circumstances (Krugman 2024a). Observers point to political partisanship and residual effects of the pandemic as contributing to the purported gap between objective indicators and subjective perceptions (Krugman 2024a; Wallace-Wells 2023).

The puzzles surrounding this potentially potent set of bad vibes (Scanlon 2022) about the broader economy inspired us to consider whether and how these perceptions might influence other social-psychological dynamics. In particular, we wondered how (if at all) perceptions of the broader economy shape the relationship between individuals’ personal experience of financial strain and their sense of powerlessness.

Sociologists have empirically established a positive association between financial strain and powerlessness (Pearlin et al. 1981). Research has also explored how objective indicators of the economy (e.g., rates of unemployment) moderate the negative association between stressors such as job insecurity and mental health outcomes (Clark 2003; Lam, Fan, Moen 2014; Turner 1995). However, while individuals’ perceptions of broader economic conditions are either implicitly or explicitly theorized to be a mechanism behind observed effects, few studies measure individuals’ perceptions of the broader economy directly. This body of research has also produced mixed findings, with scholars calling for cross-national comparisons between the United States and Canada as a potential way to reconcile them (Glavin and Young 2017).

In this study, we address these previous gaps in the literature. We first replicate the well-established positive association between financial strain and the sense of powerlessness and then ask two questions:

*Research Question 1:* How do perceptions of the economy moderate the association between financial strain and the sense of powerlessness?
*Research Question 2:* Do any observed moderation effects differ between the United States and Canada?

To address these questions, we analyze data from two nationally representative surveys of American and Canadian workers in the latter half of 2023.

## Background

### Financial Strain and Powerlessness

*Financial strain* refers to “the difficulties that individuals experience in paying bills and acquiring the basic necessities of life, such as food, clothing, housing, and medical care” (Pudrovska et al. 2005:636). In the sociology of mental health literature, scholars conceptualize financial strain as a chronic stressor that can have deleterious consequences for psychological well-being (Bierman et al. 2023; Kahn and Pearlin 2006; Pearlin et al. 1981; Wilson et al. 2020).

In the present study, we are interested in the focal association between financial strain and the sense of powerlessness, which refers to individuals’ perceived lack of personal control over the events and outcomes in everyday life (Wheaton 1983). As Seeman (1959:784) defines it in his classic conceptualization of alienation, *powerlessness* refers to the “expectancy or probability held by the individual that [their] own behavior cannot determine the occurrence, or reinforcements, [they seek].” Scholars conceptualize powerlessness as the inverse of the sense of personal control—which, like mastery, locus of control, self-efficacy, and other similar constructs—measures the belief that life's outcomes can be proactively managed by one's actions (Brett and Dubash 2023; Pearlin and Schooler 1978). Ross and Mirowsky (2012), in their extensive discussion of the relationships among these related constructs, observe that powerlessness is often a subjective reflection of the lack of objective power that individuals possess. Because financial strain is characterized by a lack of resources and increased insecurity, its link to powerlessness makes intuitive sense (Glavin, Bierman, and Schieman 2021; Koltai, Bierman, and Schieman 2018). The potential relevance of individuals’ perceptions of the broader economy for the nature and strength of that association, however, remains unexplored.

### An Empirical Puzzle: Exacerbation or Attenuation?

Before we proceed to mapping our hypotheses and the theoretical rationales behind them, we address an empirical puzzle. Consider a hypothetical scenario in which two individuals report equally high levels of financial strain. One individual, however, perceives the broader economy as performing well (Person A); the other individual perceives it as performing poorly (Person B). If we find that Person A reports higher levels of powerlessness than Person B, the question becomes: To what extent can we infer that perceptions of a good economy exacerbate the deleterious consequences of financial strain on powerlessness—and not that perceptions of a poor economy have a protective role?

In our study, we adjudicate between these complementary hypotheses in the way we operationalize perceptions of the economy. As we detail later, we adopted a measure from the Survey of Household Economics and Decisionmaking and make comparisons between three categories: “poor,”“only fair,” and “good/excellent.” We use fair as the reference category and then compare negative (poor) and positive (good) evaluations. Note that throughout this article, when we refer to a “poor,”“fair,” or “good” economy, we are referring to individual-level perceptions of the economy unless otherwise specified. To find empirical support for the hypothesis that a poor economy has a protective function, the association between financial strain and powerlessness should be weaker for those who see the economy as poor than for those who see it as fair. Its complementary scenario proposes that a good economy exacerbates the deleterious consequences of financial strain. To find empirical support for this complementary scenario, the relationship between financial strain and powerlessness should be stronger for those who perceive a good economy than for those who perceive it as fair. It is therefore possible that we might find support for only one hypothesis and not its complementary scenario. For instance, if we find differences between individuals who perceive a poor economy and those who perceive it as fair, we would find support for the protection hypothesis. If we do not find differences, however, between individuals who perceive a good economy and those who perceive it as fair, we would not find support for the exacerbation hypothesis.

**Figure 1.**
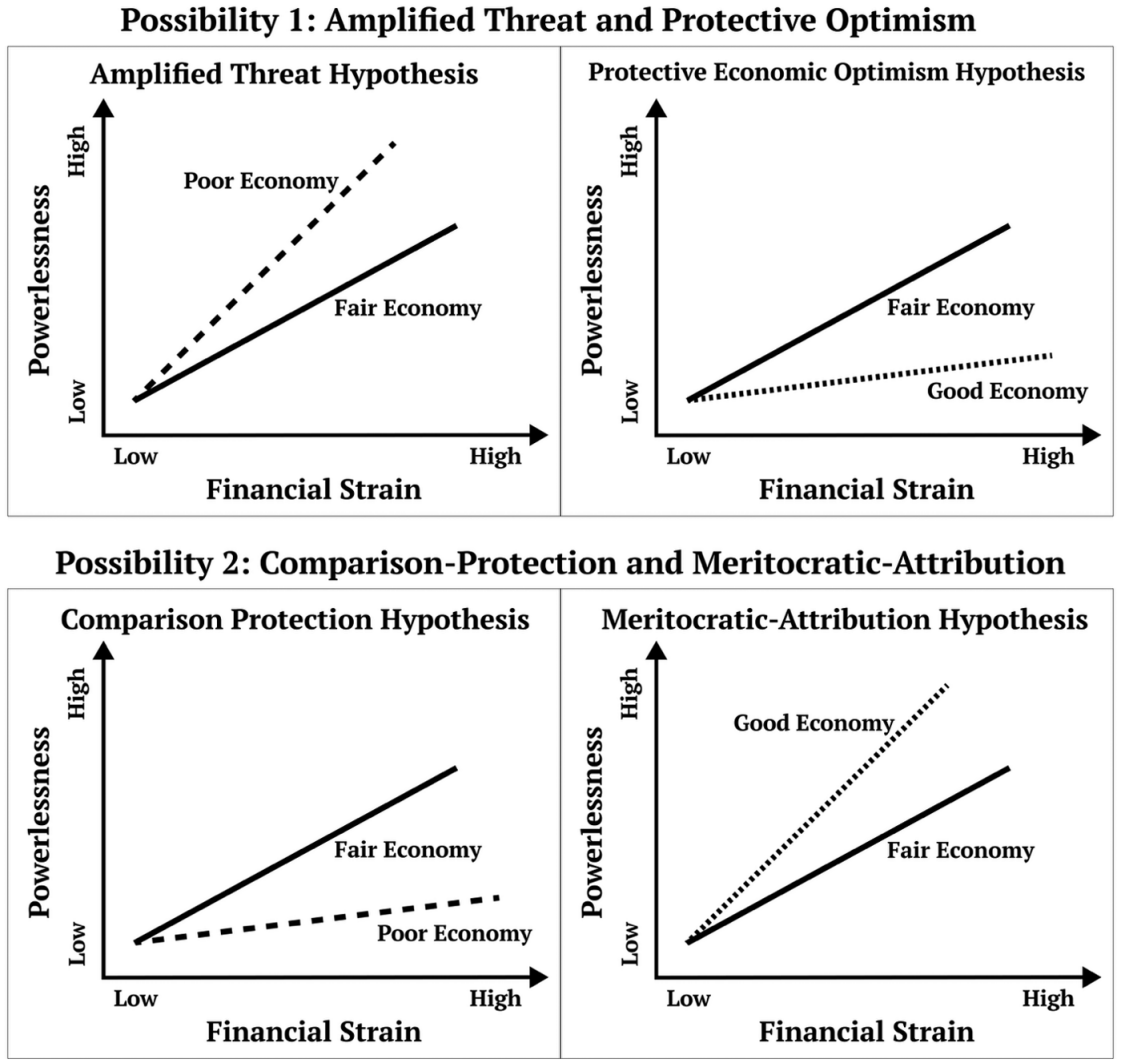
Theoretical Models *Note:* For each hypothesis, only the comparison with perceiving a fair economy is presented.

Next, we outline two competing sets of possibilities in which perceptions of the economy might moderate the relationship between financial strain and powerlessness. Each set contains two complementary scenarios. These scenarios describe what different perceptions of the economy might imply for how individuals experience their own personal financial strain. The first set of hypotheses suggest that among individuals who experience more financial strain, a poor economy is associated with more powerlessness, and a good economy is associated with less powerlessness; the second set of hypotheses proposes the opposite. Figure 1 illustrates the scenarios. It is theoretically possible for good and poor to move in the same direction relative to fair (e.g., if we find support for both the amplified threat hypothesis and the meritocratic attribution hypothesis; see Figure 1). For this reason, we focus on comparisons with a fair economy for each hypothesis. In the following sections, we describe each set of hypotheses in greater detail.

### Possibility 1: Amplified Threat and Protective Optimism Hypotheses

We first articulate complementary hypotheses about how a poor economy could be threatening and conversely, how a good economy could be protective. The “amplified threat” hypothesis predicts that the positive association between financial strain and powerlessness is stronger among individuals who perceive a poor economy; by contrast, the “protective optimism” hypothesis predicts that the positive association between financial strain and powerlessness is attenuated among individuals who perceive a good economy. Although the two hypotheses may be considered converse, we discuss them separately because they may rest on different theoretical mechanisms. Furthermore, as we established earlier, we may only find support for one but not the other.

#### Amplified threat hypothesis

We extrapolate from Glavin and Young's (2017) ideas about the interaction between personal job insecurity and the local labor market context to articulate the amplified threat hypothesis (also see Lam et al. 2014). In their study, the amplified threat hypothesis predicts that the psychological consequences of job insecurity might be worse for individuals in unfavorable labor market contexts “because of the limited opportunities for reemployment in the event of job loss” (Glavin and Young 2017:233).

We see a potential crosswalk to the ways that individuals’ perceptions of the broader economy might influence the nature of the positive association between financial strain and powerlessness. Applied here, the amplified threat hypothesis predicts that financial strain might pose an even greater threat to individuals’ sense of powerlessness if they perceive that they live in a broader economic context with scarce opportunities to improve their own financial well-being and are thus more likely to remain trapped in dealing with personal financial struggles. Aneshensel, Pearlin, and Schuler's (1993) concept of “role captivity” is potentially relevant because it argues that unfavorable financial circumstances might seem more intractable. A poor economy might dampen optimism that better days lie ahead—financially speaking. These logics suggest that the association between financial strain and powerlessness is stronger among those who perceive a poor economy compared to those who perceive a fair economy.

*Amplified economic threat hypothesis:* The positive association between financial strain and the sense of powerlessness is stronger among individuals who perceive a poor economy than those who perceive a fair economy.

Because the amplified threat hypothesis centers on financially strained individuals who perceive the economy as poor, it offers a less clearcut prediction for comparisons between fair and good economic perceptions. We address this question next by proposing the protective economic optimism hypothesis.

#### Protective economic optimism hypothesis

From an alternative angle, it is plausible that a good economy could instill a kind of protective economic optimism that attenuates the link between financial strain and powerlessness. Among individuals who are financially struggling, a good economy might offer some reassurance if it signals potential opportunities to escape their predicament. These ideas also reflect the converse of role captivity characterized by the amplified threat hypothesis. Mirowsky, Ross, and Van Willigen's (1996) concept of “American instrumentalism” provides an additional theoretical rationale for protective economic optimism. They theorize that individuals who personally “feel overwhelmed by external or random forces take heart if most others seem to manage their lives effectively” (Mirowsky et al. 1996:325). This implies a protective boost from making this form of upward social comparison if individuals perceive favorable economic conditions. In other words, a good economy may suggest to those who are financially struggling that others have some degree of effective control over their financial circumstances, which, in turn, has a reassuring effect.

Some empirical research lends support to this theoretical framing. For example, after discovering that the association between job loss and distress is absent among those living in areas with low unemployment, Turner (1995:224) concludes: “it is better to lose a job when the chances for reemployment are good.” In other words, Turner does not find evidence that losing a job has mental health costs if the person resides in an area where job loss is uncommon. Likewise, one year after the Great Recession, Lam et al. (2014) find that American workers with greater job insecurity report lower levels of happiness compared to more secure workers—but they do not find those same patterns a year before the Great Recession. These patterns suggest that being in a context where others are less likely to face job insecurity seems to be protective, thereby providing reassurance to workers facing job insecurity. The findings in both studies are consistent with the underlying ideas of the protective economic optimism hypothesis.

In sum, a good economy could attenuate the positive association between financial strain and powerlessness either because one might perceive economic opportunities as abundant or because one might, as Mirowsky et al.’s (1996) ideas suggest, believe that most others have a sense of control over the events and outcomes in their lives.

*Protective economic optimism hypothesis:* The positive association between financial strain and the sense of powerlessness is weaker among individuals who perceive a good economy than those who perceive a fair economy.

### Possibility 2: Comparison Protection and Meritocratic Attribution Hypotheses

Thus far, we have provided theoretical rationales for two complementary scenarios of the first possibility—for those who are financially struggling, a poor economy could worsen the effects of financial strain, and a good economy could dampen them. We now turn to the second possibility, which represents the oppositive line of logic: perceiving a poor economy could be advantageous, whereas a good economy might be deleterious. Following the same logic outlined earlier, we discuss these two hypotheses separately because they may rest on distinct theoretical mechanisms.

#### Comparison protection hypothesis

In opposition to the amplified threat hypothesis, the comparison protection hypothesis posits that a poor economy should weaken the positive association between financial strain and powerlessness. Social comparison theory provides a rationale for this protective effect (Festinger 1954). When facing stressful circumstances, “individuals compare themselves with similar others to discern an acceptable response . . . based on prevailing values and norms” (Young and Wheaton 2013:483). When a stressor, such as financial strain, is perceived as commonly shared by others—that is, in the context of a poor economy—its link to unfavorable psychological outcomes may be weakened because of the stressor's increased acceptability (Glavin and Young 2017; Platt and Kreitman 1985). This application of social comparison theory predicts that a poor economy should attenuate the association between financial strain and powerlessness because it might signal to individuals that they are not alone in their experience of financial strain.

Some evidence about the interplay between job insecurity and the unemployment rate supports these ideas. For example, in regions with higher unemployment rates, the unemployed and workers who experience job insecurity report more favorable levels of psychological well-being relative to their counterparts who live in regions with lower unemployment rates (Clark 2003; Cohn 1978; Flint et al. 2013; Fullerton et al. 2020; Glavin and Young 2017). These studies indicate that a high local unemployment rate has a protective effect against job loss or insecurity. In the Glavin and Young (2017:242) study, for example, in areas with higher unemployment rates, workers facing job insecurity and those not facing it report “rather comparable values” of mastery (the inverse of powerlessness). These ideas provide a rationale for the following:

*Comparison protection hypothesis:* The positive association between financial strain and the sense of powerlessness is weaker among individuals who perceive a poor economy than those who perceive a fair economy.

Because the comparison protection hypothesis centers on financially strained individuals who perceive the economy as poor, it offers a less clearcut prediction for comparisons between fair and good economic perceptions. We address this question by proposing the meritocratic attribution hypothesis.

#### Meritocratic attribution hypothesis

If a poor economy signals that individuals are not alone in struggling, then a good economy might suggest the opposite—that other people are doing better than oneself financially. Such perceptions may lead to feelings that one has been unjustly deprived of economic security compared to a similar other, resulting in personal relative deprivation (Crosby 1976). In other words, when people believe that they are denied outcomes that they deserve but comparable others receive, they may infer that factors beyond individual effort—and thus factors beyond their personal control—are determining life outcomes (Callan et al. 2024; Kraus, Piff, and Keltner 2009). Hence, experiencing relative deprivation may heighten feelings of powerlessness.

Research on meritocracy beliefs provides an alternative theoretical mechanism underlying this prediction. Beliefs that their societies are meritocratic—that is, one's success is dependent on their ability and hard work—are widely held across Western countries, and evidence suggests that these beliefs have only strengthened in recent years despite rising levels of income inequality (Mijs 2018, 2021; Mijs and Savage 2020). Such beliefs may have negative consequences for low-status groups. Political philosopher Sandel (2020:25–26) argues that meritocracy beliefs have “a corrosive effect on the way we interpret our success (or the lack of it)” and that “[f]or those who can't find work or make ends meet, it's hard to escape the demoralizing thought that their failure is their own doing, that they simply lack the talent and drive to succeed.”

These logics suggest that in societies where meritocracy beliefs are widespread, people may be more likely to attribute the cause of their failures to themselves rather than external factors, such as discrimination (McCoy and Major 2007). Applied to our case, attributing the causes of financial strain internally could exacerbate the positive association between financial strain and powerlessness. The theory of learned helplessness posits that after individuals experience a negative and uncontrollable event—or stressor—whether they learn to expect uncontrollability and generalize this expectation to other situations depends partly on the causal attribution they make (Abramson, Seligman, and Teasdale 1978). This causal attribution has three qualities: internal versus external, stable versus unstable, and global versus specific. One perspective argues that internal, stable, and global attributions tend to foster learned helplessness—a concept that Mirowsky and Ross (1986:28) argue is “roughly interchangeable” with powerlessness. In other words, individuals learn to expect uncontrollability if they interpret a stressor as being caused by themselves, if the cause is perceived as rigid and unchangeable, and if it is present across a variety of situations (Kinderman and Bentall 1996). If individuals learn to expect uncontrollability, this will likely foster a greater generalized sense of powerlessness.

Taken together, in societies with more widespread meritocracy beliefs, a good economy may signal to those who are financially struggling that their predicaments are caused by themselves instead of due to broader economic forces. The attribution of financial strain to their own actions and not the broader economy may alter their sense of whether they can control circumstances in other areas of their lives, shifting what may be the attribution of a more unstable and specific circumstance to stable and global patterns in their situation. Thus, when one experiences financial strain, a good economy may lead to internal causal attribution of this strain, which could intensify the link between financial strain and the sense of powerlessness (Glavin and Young 2017; Sweeney, Anderson, and Bailey 1986). These dynamics suggest the following:

*Meritocratic attribution hypothesis:* The positive association between financial strain and powerlessness is stronger among individuals who perceive a good economy than those who perceive a fair economy.

### Canada and United States Comparison

Our review of the literature so far shows conflicting empirical findings. Attempting to reconcile their findings with prior research, Glavin and Young (2017) posit that these differences may be a result of different national contexts. Because their study uses a sample of Canadians, the authors speculate that there may be “cultural differences in the meaning and value of employment” and raise the possibility that “Americans’ emphasis on individualism and economic mobility offsets any influence of a social comparison process” (Glavin and Young 2017:247). Echoing this speculation, Lipset (1964:175; see also Lipset 1986) argues the following:

To a very great extent Canada and the United States share the same values but . . . in Canada these values are *held much more tentatively* [italics added]. . . . [W]hile equality and achievement, for example, are values emphasized in both American societies, in Canada the emphasis is somewhat less and therefore the contrast between the nations remains one of degree.

In other work, Lipset (1990) further argues that Canadians, who emphasize equality of outcomes, tend to put less emphasis on meritocracy than Americans do. Hence, it is possible that a poor and good economy mean different things to financially struggling Americans and Canadians. For example, if Canadians do tend to put less emphasis on meritocracy, perceiving a good economy might not convey the same level of threat to those who are financially struggling as it does in the United States. Our study puts these ideas to test.

## Method

The data for this study come from two nationally representative samples of workers in the United States (N = 2,466) and Canada (N = 2,501). The Measuring Employment Sentiments and Social Inequality survey was designed to capture the sentiments and working conditions of the American working population.^1^ Similarly, the Canadian Quality of Work and Economic Life Study is a cross-sectional survey to assess the employment and economic sentiments of Canadian workers. In addition to using identical questions for all focal variables in our analyses, both surveys recruited participants using existing online panels during a similar time period (September 2023 in Canada and November 2023 in the United States), making them ideal samples to compare. Both surveys were conducted online through professional research firms, Angus Reid Group in Canada and YouGov in the United States, who maintain panels of respondents and selectively sample to represent the demographic characteristics of the national working population. The sampling means for each survey are reported in Table 1.

**Table 1.** Descriptive Statistics

Variables	United States (*N* = 2,466)	Canada (*N* = 2,501)
Powerlessness	2.448 (.676)	2.317 (.730)
Financial strain	1.449 (1.081)	1.404 (1.067)
Economy		
Poor	.383	.475
Fair	.399	.378
Good	.218	.146
Bachelor’s degree		
No	.550	.633
Yes	.450	.367
Age	44.072 (13.803)	44.168 (12.960)
United States: party identification		
Republican	.260	—
Democrat	.352	—
Independent	.286	—
Other	.039	—
Not sure	.064	—
Canada: party voted for		
Did not vote	—	.104
Conservative	—	.265
Liberal	—	.250
New Democratic Party	—	.208
Other	—	.112
Unknown	—	.060
Household income		
Less than 50,000	.215	.168
50,000 to 100,000	.402	.332
100,000 or more	.308	.417
Don’t know	.075	.082
Professional		
No	.573	.707
Yes	.427	.294
Cohabitation		
No	.440	.327
Yes	.560	.673
White		
No	.366	.160
Yes	.634	.841
Gender		
Men	.515	.523
Women	.485	.477

*Note:* Unweighted. Proportions are presented for categorical variables. Standard deviations are in parentheses.

### Variables

#### Powerlessness

To operationalize powerlessness, we used four items: (1) “You have little control over the things that happen to you,” (2) “There is really no way you can solve some of the problems you have,” (3) “You often feel helpless in dealing with problems of life,” and (4) “Sometimes you feel that you are being pushed around in life.” The response choices range from 1 = strongly agree to 4 = strongly disagree. We reversed-coded and took the mean of these four items. A higher score represents higher powerlessness (Cronbach’s α = .831 for the American sample; α = .835 for the Canadian sample). These items come from the Pearlin Mastery Scale (Pearlin and Schooler 1978); we refer to the underlying construct as powerlessness here, instead of mastery or personal control, because these items only capture respondents’ perceived lack of control and do not capture the sense of control they perceive over good and bad outcomes in their lives (see Mirowsky and Ross 2003).

#### Financial strain

To operationalize financial strain (see Koltai et al., 2018), we took the mean of three items: (1) “How often in the past year did you have trouble paying the bills?”; (2) “How often in the past year did you not have enough money to buy food, clothes or other things your household needed?”; and (3) “How do your finances usually work out by the end of the month? Would you say you have . . . .” Response choices for the first two items include 0 = never, 1 = rarely, 2 = sometimes, 3 = often, and 4 = very often. Response choices for the third question include 0 = a lot of money left over, 1 = a little money left over, 2 = just enough to make ends meet, 3 = barely enough to get by, and 4 = not enough to make ends meet. A higher score represents higher financial strain (α = .838 for the American sample; α = .863 for the Canadian sample).

#### Perceptions of the economy

To measure perceptions of the economy, we asked: “In this country, how would you rate economic conditions today?” This measure is adopted from the 2022 Survey of Household Economics and Decisionmaking survey. We provided four response choices: “poor,”“only fair,”“good,” and “excellent.” We combined good and excellent (due to the small cell size of the excellent category; N = 53 for the American sample and N = 26 for the Canadian sample), and we label this group as “good.” For brevity, furthermore, we generally refer to the only fair group as “fair” throughout the article.

#### Covariates

We include a range of covariates, including whether the respondent holds a bachelor's degree, their age, household income, whether they work in a professional job, whether they are cohabitating with another person, whether they are white, and their gender. We also control for political party identification in the United States and the political party respondents voted for in the last federal election in Canada because perceptions of the economy are deeply intertwined with political orientation (Krugman 2024a); however, results are substantively the same with or without controlling for these two variables.

### Analytical Plan

We use linear regression models to test our hypotheses. Model 1 regresses powerlessness on perceptions of the economy and financial strain along with covariates. Model 2 adds an interaction term between economy perceptions and financial strain. We run separate regression models for the American and the Canadian samples.

## Results

Table 2 presents the results of regressing powerlessness on financial strain and perceptions of the economy in both the United States and Canada using fair as the reference category for perceptions of the economy. We begin by examining the net effects of economic perceptions in Model 1. Model 1a and Model 1b show that in both samples, financial strain is positively associated with powerlessness (United States: *b* = .248, *p* < .001; Canada: *b* = .245, *p* < .001). In both the United States and Canada, furthermore, the association between perceptions of the economy and powerlessness is not statistically significant.

**Table 2.** Powerlessness Regressed on Financial Strain and Perceptions of the Economy (Reference: Fair)

	United States (*N* = 2,466)		Canada (*N* = 2,501)	
Variables	Model 1a	Model 2a	Model 1b	Model 2b
Economy (reference = fair)				
Poor	.042 (.030)	.099* (.050)	.020 (.029)	.087 (.048)
Good	.018 (.034)	−.104 (.058)	.017 (.041)	−.089 (.063)
Financial strain	.248*** (.014)	.245*** (.023)	.245*** (.014)	.257*** (.022)
Financial strain × economy				
Financial strain × poor		−.032 (.029)		−.042 (.028)
Financial strain × good		.099** (.037)		.108* (.047)
Bachelor’s degree	.032 (.030)	.031 (.030)	−.030 (.030)	−.029 (.030)
Age	−.008*** (.001)	−.008*** (.001)	−.011*** (.001)	−.010*** (.001)
Household income (reference = 50,000–100,000)				
Less than 50,000	.059 (.036)	.061 (.036)	−.013 (.040)	−.009 (.041)
100,000 or more	−.028 (.033)	−.024 (.033)	−.002 (.032)	−.003 (.032)
Don’t know	.062 (.050)	.057 (.050)	.077 (.052)	.079 (.052)
United States: party identification (reference = Democrat)				
Republican	−.056 (.038)	−.062 (.037)	—	—
Independent	.003 (.032)	.008 (.032)	—	—
Other	−.094 (.065)	−.086 (.065)	—	—
Not sure	.075 (.053)	.078 (.052)	—	—
Canada: party voted for (reference = Liberal)				
Did not vote	—	—	−.047 (.051)	−.048 (.051)
Conservative	—	—	.015 (.038)	.006 (.038)
New Democratic Party	—	—	.067 (.039)	.067 (.039)
Other	—	—	.070 (.049)	.062 (.049)
Unknown	—	—	−.161* (.062)	−.165** (.061)
Professional	−.013 (.031)	−.009 (.031)	−.052 (.031)	−.047 (.031)
Cohabitation	.011 (.028)	.007 (.028)	−.069* (.031)	−.070* (.031)
White	.088** (.028)	.086** (.028)	−.112** (.037)	−.111** (.037)
Women	.003 (.026)	.009 (.026)	.049 (.027)	.047 (.027)
Constant	2.342*** (.064)	2.334*** (.065)	2.559*** (.078)	2.530*** (.081)
*R* ^2^	.235	.241	.207	.211

*Note:* Weighted analysis. Standard errors are in parentheses.

* *p* < .05. **p < .01. ***p < .001.

**Figure 2.**
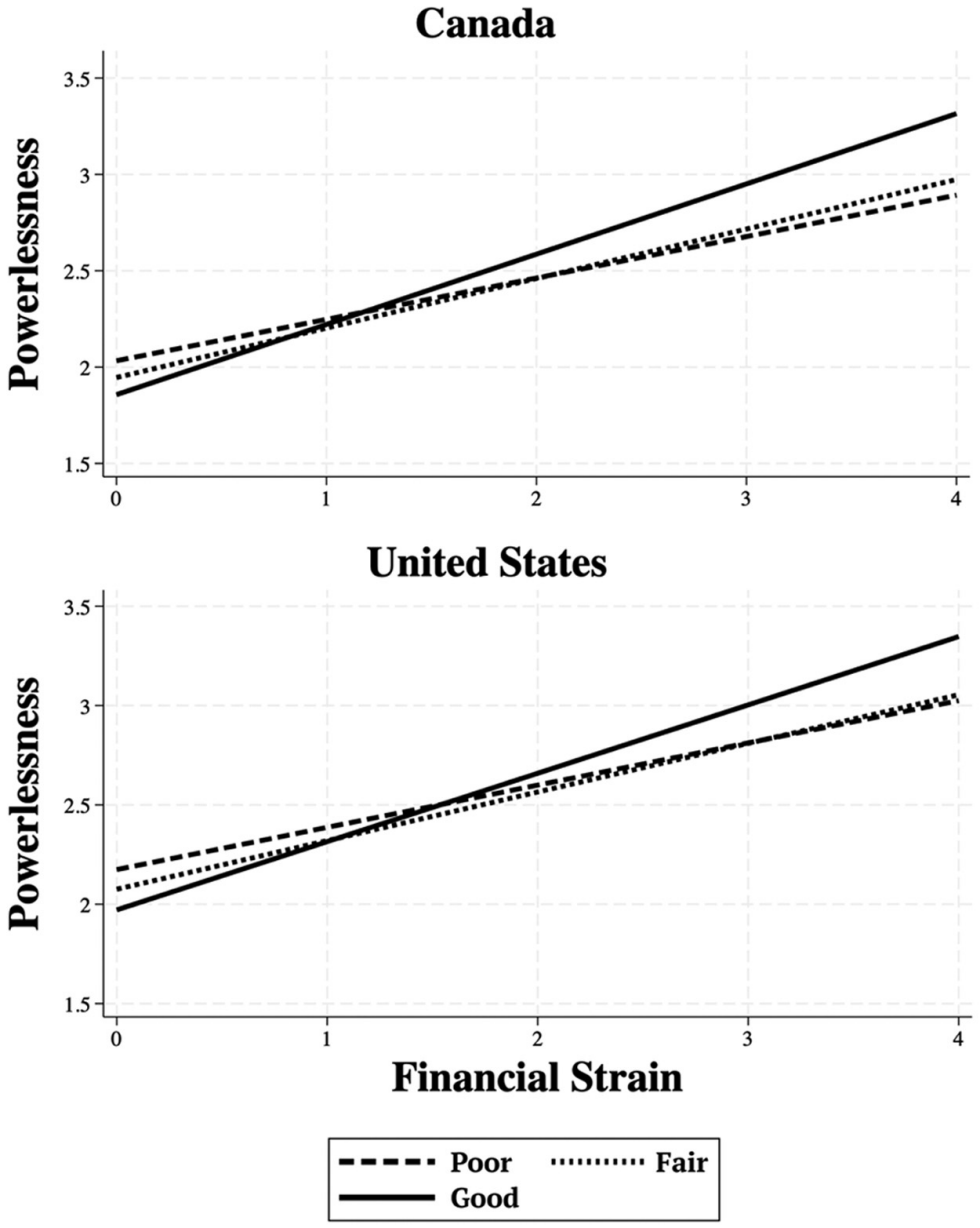
The Relationship between Financial Strain and Powerlessness by Perceptions of the Economy

Model 2 tests the interaction between financial strain and perceptions of the economy. The interaction effects in the two samples are similar. In both the United States and Canada, compared to those who perceive a fair economy, the positive association between financial strain and powerlessness is stronger for those who perceive a good economy (United States: *b* = .099, *p* < .01; Canada: *b* = .108, *p* < .05). Table 2 also shows no statistically significant differences in the association between financial strain and powerlessness when comparing poor to fair (United States: *b* = −.032, *p* > .05; Canada: *b* = −.042, *p* > .05). These effects are presented in Figure 2, with the Canadian results in the top panel and the American results in the bottom panel.

In Table 3, we switch the reference category to the perception of a poor economy and confirm that the association between financial strain and powerlessness is stronger for those who perceive a good economy versus those who perceive a poor economy (United States: *b* = .131, *p* < .001; Canada: *b* = .150, *p* < .01). Taken together, the significant interaction effects in both countries are consistent with the meritocratic attribution hypothesis.

**Table 3.** Powerlessness Regressed on Financial Strain and Perceptions of the Economy (Reference: Poor)

	United States (N = 2,466)		Canada (N = 2,501)	
Variables	Model 1a	Model 2a	Model 1b	Model 2b
Economy (reference = poor)				
Fair	−.042 (.030)	−.099* (.050)	−.020 (.029)	−.087 (.048)
Good	−.024 (.037)	−.203** (.062)	−.003 (.042)	−.176** (.066)
Financial strain	.248*** (.014)	.213*** (.020)	.245*** (.014)	.215*** (.019)
Financial strain × economy				
Financial strain × fair		.032 (.029)		.042 (.028)
Financial strain × good		.131*** (.035)		.150** (.046)

*Note:* Weighted analysis. Standard errors are in parentheses. Controls for all covariates in Table 2.

* *p* < .05. **p < .01. ***p < .001.

Model 2a and 2b also reveal some main effects that are worth discussing. Among individuals who do not experience any financial strain, those who perceive a good economy report lower levels of powerlessness than those who perceive a poor economy in both samples (Table 3; United States: *b* = −.203, *p* < .001; Canada: *b* = −.176, *p* < .01). Table 3 also shows that those who perceive a fair economy report lower levels of powerlessness than those who perceive a poor economy in the United States when financial strain equals zero (*b* = −.099, *p* < .05), but this difference is not significant at the .05 level in Canada (*b* = −.087, *p* = .072). Lastly, Model 2a and 2b in Table 2 show that in both the United States and Canada, those who perceive a good economy do not report statistically different levels of powerlessness compared to those who perceive a fair economy (United States: *b* = −.104, *p* > .05; Canada: *b* = −.089, *p* > .05) when financial strain equals zero.

## Discussion

We have argued that perceptions of the economy might alter the meanings individuals attribute to their financial struggles, presenting two competing perspectives on how that could shape the relationship between financial strain and powerlessness. The first perspective proposes that (a) a poor economy could amplify perceptions of financial threat to individuals’ already vulnerable financial position and that (b) a good economy could ameliorate negative effects of financial strain through hope in their prospects. The second perspective proposes the opposite—(a) a poor economy could be reassuring because it signals to individuals who are struggling financially that others are struggling as well, whereas (b) a good economy could intensify the powerlessness of financial strain through experiences of relative deprivation and internal causal attribution.

We find support for the second perspective in both the United States and Canada—but only the (b) side of that argument. Those who are financially struggling and perceive a good economy experience more powerlessness on average than those who perceive a fair or poor economy. We do not find statistically significant differences in the association between financial strain and powerlessness between those who perceive a poor economy and those who perceive a fair economy. In short, relative to those who perceive the economy as fair or poor, we find evidence that perceiving a good economy while financially struggling is associated with greater powerlessness. By contrast, we do not find evidence that perceptions of a poor economy make financial strain's association with powerlessness weaker relative to perceptions of a fair economy.

Among those who perceive a good economy, two underlying mechanisms could be operating: relative deprivation and internal causal attribution. On one hand, financially struggling in the context of a (perceived) good economy may elevate the sense of being unjustly deprived of financial security; alternatively, under a backdrop of widespread meritocracy beliefs, financially struggling in the context of a (perceived) good economy may encourage the attribution of that financial strain to internal causes (e.g., one's character or actions).

Our study makes several contributions. Whereas prior research has examined how objective economic indicators at the national and local levels can moderate the relationship between stressors and mental health outcomes, we use subjective perceptions of the broader national economy. Although the struggle to afford basic necessities has an objective reality with adverse consequences for many, economic perceptions have an effect of their own that may be distinct from the objective reality of the broader economy (Krugman 2024b). We find that using a subjective measure yields different conclusions from prior research. For example, Glavin and Young (2017), in their study of the moderating role of local unemployment rates, suggest that perceiving others as being “in the same boat” when facing job insecurity may have a protective effect. Extrapolating these findings to our case suggests that we should find support for the comparison protection hypothesis—that the positive association between financial strain and powerlessness should be weaker for those who perceive a poor economy than those who perceive a fair economy. Our study, however, finds evidence that for financially struggling individuals, perceiving a good economy elevates levels of powerlessness, and we do not find evidence that perceiving a poor economy attenuates it, compared to those who perceive a fair economy.

One possible explanation for these diverging patterns could be that perceptions of economic circumstances and objective economic conditions moderate the relationship between stressors and mental health outcomes through different mechanisms. Glavin and Young (2017:235) suggest that their findings might also be explained by a perceived sense of social support: “individuals surrounded by similar others may assume a sense of shared empathy, emotional support, and generalized social support.” Hence, it is possible that worsened objective economic circumstances (e.g., high local unemployment rate) may be protective through activating factors such as social support rather than through individuals’ social comparison processes. This could at least partially explain the divergent findings between our study and that of Glavin and Young's study.

An alternative interpretation of the conflicting findings between our study and prior research could relate to the distinction between the local economy and the national economy (Niemi, Bremer, and Heel 1999). People who perceive the national economy as doing poorly might still perceive their local economy as doing well—and as contemporary discourse suggests, many do (e.g., Krugman 2024a). One's perceptions of their local economy might matter differently, than their perceptions of the national economy because of the immediacy and visibility of the local economy (see, however, Lee and Pescosolido 2024, for a discussion of when national economic conditions may matter more than local economic conditions). Evaluations of the local economy might be more impactful on one's perceived quality of economic life than evaluations of the national economy. When one is financially struggling, positive evaluations of the local economy might therefore be protective because they signal opportunities. We acknowledge that these distinctions are challenging to test empirically, and our study certainly does not have the capacity to address them. We offer this speculation as a possibility for future research to further explore.

Because our data are collected in 2023, it is also possible that our findings are limited to this specific historical period—a period following the COVID-19 pandemic and that some have argued to be dominated by “bad vibes” in economic perceptions (Scanlon 2022). The “vibecession” may be a recent phenomenon: For instance, 50 percent of Americans rated the national economy as either good or excellent in 2019 (Thompson 2022) compared to about 25 percent of Americans in 2023 (Casselman and DePillis 2024). How perceptions of the economy moderate the relationship between financial stressors and mental health may differ when the majority believes that the economy is well-performing compared to when the majority believes that it is poor-performing. Alternatively, it is also possible that our findings reflect the fact that (perceptions of) the performance of the economy may be a salient issue in 2023 compared to other time periods. If one does not hold an opinion of how the economy is performing (perhaps because of a lack of media attention on the issue), then we would not expect the variable to systematically influence the relationship between personal financial strain and powerlessness. Future research could examine whether our findings hold across different historical contexts or periods of economic volatility.

Another contribution of our study is that we respond to prior calls from researchers to evaluate potential cross-national differences. Our study compares two national surveys of workers in the United States and Canada collected in 2023 during a peak period of widespread media chatter about the state of the broader economy. We observe remarkably similar patterns in both countries during this period. If broad societal beliefs in meritocracy underlie the patterns we observe, the extent to which Canada and the United States differ on these beliefs seems to matter little for how perceptions of the economy moderate the association between financial strain and powerlessness. It is also possible that the United States and Canada have grown more similar with regards to meritocracy beliefs since Lipset’s (1964, 1990) writings. We are limited by our data to test these possibilities directly, and future research could replicate our studies in countries where meritocracy beliefs are not as deeply ingrained.

Before concluding, we wish to cite a few limitations. First, although we have cited the potential relevance of relative deprivation and attribution theories in our interpretations of how perceptions of the economy moderate the relationship between financial strain and powerlessness, we are unable to adjudicate specific underlying mechanisms within those processes. Second, we are limited by our cross-sectional data; future research could use longitudinal analyses to minimize omitted variable bias that might be influential in our observations. Third, due to the small cell sizes of the excellent category for the perceptions of the economy variable, we could not compare those who perceived the economy as excellent to others. Fourth, although we use the terms “powerlessness” and “financial strain” because they are among the most prevalent in the sociology of mental health, they overlap with related constructs to a great extent. Powerlessness overlaps considerably with constructs such as external locus of control, learned helplessness, and a lack of self-efficacy (Ross and Mirowsky 2012), and financial strain overlaps with economic hardship, financial worry, economic scarcity, and financial security (Williamson and Munyon 2025). Future research could aim to systematically compare these constructs to allow further comparability across this and related studies findings. Finally, the findings presented here are associative in nature and should not be interpreted causally. For instance, it is possible that, at least to some degree, individuals’ sense of powerlessness might influence their perceptions of the economy.

Despite these limitations, our study makes an important contribution to the ongoing discussion of the importance of perceptions of the economy and its role in shaping the quality of individuals’ lives. As recent discourse about the disconnect between the objective and subjective aspects of broader economic conditions indicate, perceptions may matter more than objective indicators. But the empirical twist in our study is that perceptions of a good economy may be more problematic—at least as it relates to the link between financial strain and powerlessness. As broader economic conditions deteriorate or improve in the United States and Canada, it will be interesting to track how the social-psychological dynamics underlying the patterns we documented might also change over time.

## Notes

1 Note that the full Measuring Employment Sentiments and Social Inequality 2023 sample includes 5,000 respondents; here, we analyze data from a randomized half assigned to the module that includes the question on economic perceptions.

## References

[bibr1-01902725261421204] Abramson Lyn Seligman Martin Teasdale John . 1978. “Learned Helplessness in Humans: Critique and Reformulation.”Journal of Abnormal Psychology 87(1):49–74. 649856

[bibr2-01902725261421204] Amanat Hayatullah . 2023. “Most Canadians Pessimistic about Canada's Economy, Survey Finds.”CTV News, January 25. https://vancouverisland.ctvnews.ca/lifestyle/article/most-canadians-pessimistic-about-canadas-economy-survey-finds/.

[bibr3-01902725261421204] Aneshensel Carol S. Pearlin Leonard I. Schuler Roberleigh H. 1993. “Stress, Role Captivity, and the Cessation of Caregiving.”Journal of Health and Social Behavior 34(1):54–70. 8463635

[bibr4-01902725261421204] Bierman Alex Upenieks Laura Lee Yeonjung Harmon Megan . 2023. “Consequences of Financial Strain for Psychological Distress among Older Adults: Examining the Explanatory Role of Multiple Components of the Self-Concept.”Socius 9. doi:10.1177/23780231231197034.

[bibr5-01902725261421204] Brett Gordon Dubash Soli . 2023. “The Sociocognitive Origins of Personal Mastery.”Journal of Health and Social Behavior 64(3):452–68. 10.1177/00221465231167558 PMC10486156 37129297

[bibr6-01902725261421204] Callan Mitchell J. Sutton Robbie M. Chobthamkit Phatthanakit Yeung Victoria Wai Lan Leung Florence Y. N. Asano Ryosuke Beattie Peter , et al. 2024. “Personal Relative Deprivation and Locus of Control.”Journal of Personality 93(4):845–65. 10.1111/jopy.12980 PMC12224560 39435651

[bibr7-01902725261421204] Casselman Ben DePillis Lydia . 2024. “Brighter Economic Mood Isn't Translating into Support for Biden.”The New York Times, March 5. https://www.nytimes.com/2024/03/05/business/economy/biden-economy-times-siena-poll.html.

[bibr8-01902725261421204] Clark Andrew E. 2003. “Unemployment as a Social Norm: Psychological Evidence from Panel Data.”Journal of Labor Economics 21(2):323–51.

[bibr9-01902725261421204] Cohn Richard M. 1978. “The Effect of Employment Status Change on Self-Attitudes.”Social Psychology 41(2):81–93. doi:10.2307/3033568.

[bibr10-01902725261421204] Crosby Faye . 1976. “A Model of Egoistical Relative Deprivation.”Psychological Review 83(2):85–113.

[bibr11-01902725261421204] Festinger Leon . 1954. “A Theory of Social Comparison Processes.”Human Relations 7(2):117–40.

[bibr12-01902725261421204] Flint Ellen Shelton Nicola Bartley Mel Sacker Amanda . 2013. “Do Local Unemployment Rates Modify the Effect of Individual Labour Market Status on Psychological Distress?” Health & Place 23:1–8. 23727618 10.1016/j.healthplace.2013.04.004

[bibr13-01902725261421204] Fullerton Andrew S. McCollum Destinee B. Dixon Jeffrey C. Anderson Kathryn Freeman . 2020. “The Insecurity Gradient in Health: How Inequality in the Distribution of Job Insecurity Matters for the Relationship between Job Insecurity and Self-Rated Health in Europe.”The Sociological Quarterly 61(1):107–27.

[bibr14-01902725261421204] Glavin Paul Bierman Alex Schieman Scott . 2021. “Über-Alienated: Powerless and Alone in the Gig Economy.”Work and Occupations 48(4):399–431.

[bibr15-01902725261421204] Glavin Paul Young Marisa . 2017. “Insecure People in Insecure Places: The Influence of Regional Unemployment on Workers’ Reactions to the Threat of Job Loss.”Journal of Health and Social Behavior 58(2):232–51. 10.1177/0022146517696148 28661783

[bibr16-01902725261421204] Kahn Joan R. Pearlin Leonard I. 2006. “Financial Strain over the Life Course and Health among Older Adults.”Journal of Health and Social Behavior 47(1):17–31. 16583773 10.1177/002214650604700102

[bibr17-01902725261421204] Kinderman Peter Bentall Richard P. 1996. “A New Measure of Causal Locus: The Internal, Personal and Situational Attributions Questionnaire.”Personality and Individual Differences 20(2):261–64.

[bibr18-01902725261421204] Koltai Jonathan Bierman Alex Schieman Scott . 2018. “Financial Circumstances, Mastery, and Mental Health: Taking Unobserved Time-Stable Influences into Account.”Social Science & Medicine 202:108–16. 10.1016/j.socscimed.2018.01.019 29522902

[bibr19-01902725261421204] Kraus Michael W. Piff Paul K. Keltner Dacher . 2009. “Social Class, Sense of Control, and Social Explanation.”Journal of Personality and Social Psychology 97(6):992–1004. 19968415 10.1037/a0016357

[bibr20-01902725261421204] Krugman Paul . 2024a. “Good Economy, Negative Vibes: The Story Continues.”The New York Times, April 8. https://www.nytimes.com/2024/04/08/opinion/economy-vibes.html.

[bibr21-01902725261421204] Krugman Paul . 2024b. “Is the Vibecession Finally Coming to an End?”The New York Times, January 22. https://www.nytimes.com/2024/01/22/opinion/biden-trump-vibecession-economy.html.

[bibr22-01902725261421204] Lam Jack Fan Wen Moen Phyllis . 2014. “Is Insecurity Worse for Well-Being in Turbulent Times? Mental Health in Context.”Society and Mental Health 4(1):55–73. 25436177 10.1177/2156869313507288 PMC4244885

[bibr23-01902725261421204] Lee Byungkyu Pescosolido Bernice A. 2024. “Misery Needs Company: Contextualizing the Geographic and Temporal Link between Unemployment and Suicide.”American Sociological Review 89(6):1104–40.

[bibr24-01902725261421204] Lipset Seymour Martin . 1964. “Canada and the United States-A Comparative View.”Canadian Review of Sociology / Revue Canadienne de Sociologie 1(4):173–85.

[bibr25-01902725261421204] Lipset Seymour Martin . 1986. “Historical Traditions and National Characteristics: A Comparative Analysis of Canada and the United States.”Canadian Journal of Sociology / Cahiers Canadiens de Sociologie 11(2):113–55.

[bibr26-01902725261421204] Lipset Seymour Martin . 1990. Continental Divide: The Values and Institutions of the United States and Canada. New York, NY: Routledge.

[bibr27-01902725261421204] McCoy Shannon K. Major Brenda . 2007. “Priming Meritocracy and the Psychological Justification of Inequality.”Journal of Experimental Social Psychology 43(3):341–51.

[bibr28-01902725261421204] Mijs Jonathan J. B. 2018. “Visualizing Belief in Meritocracy, 1930–2010.”Socius 4. doi:10.1177/2378023118811805.

[bibr29-01902725261421204] Mijs Jonathan J. B. 2021. “The Paradox of Inequality: Income Inequality and Belief in Meritocracy Go Hand in Hand.”Socio-Economic Review 19(1):7–35.

[bibr30-01902725261421204] Mijs Jonathan J. B. Savage Mike . 2020. “Meritocracy, Elitism and Inequality.”The Political Quarterly 91(2):397–404.

[bibr31-01902725261421204] Mirowsky John Ross Catherine E. 1986. “Social Pattern of Distress.”Annual Review of Sociology 12:23–45.

[bibr32-01902725261421204] Mirowsky John Ross Catherine E. 2003. Social Causes of Psychological Distress. Hawthorne, NY: Aldine De Gruyter.

[bibr33-01902725261421204] Mirowsky John Ross Catherine E. Van Willigen Marieke . 1996. “Instrumentalism in the Land of Opportunity: Socioeconomic Causes and Emotional Consequences.”Social Psychology Quarterly 59(4):322–37.

[bibr34-01902725261421204] Niemi Richard G. Bremer John Heel Michael . 1999. “Determinants of State Economic Perceptions.”Political Behavior 21:175–93.

[bibr35-01902725261421204] Pearlin Leonard I. Menaghan Elizabeth G. Lieberman Morton A. Mullan Joseph T. 1981. “The Stress Process.”Journal of Health and Social Behavior 22(4):337–56. 7320473

[bibr36-01902725261421204] Pearlin Leonard I. Schooler Carmi . 1978. “The Structure of Coping.”Journal of Health and Social Behavior 19(1):2–21. 649936

[bibr37-01902725261421204] Platt Stephen Kreitman Norman . 1985. “Parasuicide and Unemployment among Men in Edinburgh 1968–82.”Psychological Medicine 15(1):113–23. 10.1017/s0033291700020973 3873081

[bibr38-01902725261421204] Pudrovska Tetyana Schieman Scott Pearlin Leonard I. Nguyen Kim . 2005. “The Sense of Mastery as a Mediator and Moderator in the Association between Economic Hardship and Health in Late Life.”Journal of Aging and Health 17(5):634–60. 10.1177/0898264305279874 16177454

[bibr39-01902725261421204] Ross Catherine E. Mirowsky John . 2012. “The Sense of Personal Control: Social Structural Causes and Emotional Consequences.” Pp. 379–402 in Handbook of the Sociology of Mental Health, edited by Aneshensel C. S. Phelan J. C. Dordrecht A. Bierman . Netherlands: Springer Netherlands.

[bibr40-01902725261421204] Sahm Claudia . 2023. “Americans Like Sharing Bad Economic News Way Too Much.”Bloomberg, October 30. https://www.bloomberg.com/opinion/articles/2023-10-30/americans-like-sharing-bad-economic-news-way-too-much.

[bibr41-01902725261421204] Sandel Michael J. 2020. The Tyranny of Merit: What's Become of the Common Good? New York, NY: Farrar, Straus and Giroux.

[bibr42-01902725261421204] Scanlon Kyla . 2022. “The Vibes in the Economy Are . . . Weird. Really Weird.”The New York Times, August 4. https://www.nytimes.com/2022/08/04/opinion/us-economy-recession-federal-reserve.html.

[bibr43-01902725261421204] Schieman Scott Wilson Alexander Liang Jiarui . 2023. “Canadians Are Losing Faith in the Economy — And It's Affecting Their Perception of Inequality.”The Conversation, December 22. https://theconversation.com/canadians-are-losing-faith-in-the-economy-and-its-affecting-their-perception-of-inequality-219794.

[bibr44-01902725261421204] Seeman Melvin . 1959. “On the Meaning of Alienation.”American Sociological Review 24(6):783–91.

[bibr45-01902725261421204] Sweeney Paul D. Anderson Karen Bailey Scott . 1986. “Attributional Style in Depression: A Meta-analytic Review.”Journal of Personality and Social Psychology 50(5):974–91. 10.1037//0022-3514.50.5.974 3712233

[bibr46-01902725261421204] Thompson Derek . 2022. “Everything Is Terrible, but I'm Fine.”The New York Times, June 1. https://www.theatlantic.com/newsletters/archive/2022/06/american-economy-negative-perception-inflation/661149/.

[bibr47-01902725261421204] Turner J. Blake . 1995. “Economic Context and the Health Effects of Unemployment.”Journal of Health and Social Behavior 36(3):213–29. 7594355

[bibr48-01902725261421204] Wallace-Wells David . 2023. “Why Is It a Surprise That America Is Gloomy after a Devastating Pandemic?”The New York Times, December 6. https://www.nytimes.com/2023/12/06/opinion/bad-economy-vibes.html.

[bibr49-01902725261421204] Wheaton Blair . 1983. “Stress, Personal Coping Resources, and Psychiatric Symptoms: An Investigation of Interactive Models.”Journal of Health and Social Behavior 24(3):208–29. 6630976

[bibr50-01902725261421204] Williamson Gavin Munyon Timothy . 2025. “Financial Security Spirals at Work: A Review, Integration, and Agenda for Intervention.”Human Resource Management Review 35(3):101086. doi:10.1016/j.hrmr.2025.101086.

[bibr51-01902725261421204] Wilson Jenna M. Lee Jerin Fitzgerald Holly N. Oosterhoff Benjamin Sevi Bariş Natalie J. Shook . 2020. “Job Insecurity and Financial Concern during the COVID-19 Pandemic Are Associated with Worse Mental Health.”Journal of Occupational & Environmental Medicine 62(9):686–91. 10.1097/JOM.0000000000001962 32890205

[bibr52-01902725261421204] Young Marisa Wheaton Blair . 2013. “The Impact of Neighborhood Composition on Work-Family Conflict and Distress.”Journal of Health and Social Behavior 54(4):481–97. 10.1177/0022146513504761 24311757

